# Does motivation matter in upper-limb rehabilitation after stroke? ArmeoSenso-Reward: study protocol for a randomized controlled trial

**DOI:** 10.1186/s13063-017-2328-2

**Published:** 2017-12-02

**Authors:** Mario Widmer, Jeremia P. Held, Frieder Wittmann, Olivier Lambercy, Kai Lutz, Andreas R. Luft

**Affiliations:** 1 Division of Vascular Neurology and Neurorehabilitation, Department of Neurology, University Hospital of Zurich, Zurich, Switzerland; 2 cereneo, Center for Neurology and Rehabilitation, Vitznau, Switzerland; 3 Neural Control of Movement Laboratory, ETH Zurich, Zurich, Switzerland; 4 Rehabilitation Engineering Laboratory, ETH Zurich, Zurich, Switzerland

**Keywords:** Rehabilitation, Virtual reality, Stroke, Upper extremity, Arm, Feedback, Reward

## Abstract

**Background:**

Fifty percent of all stroke survivors remain with functional impairments of their upper limb. While there is a need to improve the effectiveness of rehabilitative training, so far no new training approach has proven to be clearly superior to conventional therapy. As training with rewarding feedback has been shown to improve motor learning in humans, it is hypothesized that rehabilitative arm training could be enhanced by rewarding feedback. In this paper, we propose a trial protocol investigating rewards in the form of performance feedback and monetary gains as ways to improve effectiveness of rehabilitative training.

**Methods:**

This multicentric, assessor-blinded, randomized controlled trial uses the ArmeoSenso virtual reality rehabilitation system to train 74 first-ever stroke patients (< 100 days post stroke) to lift their impaired upper limb against gravity and to improve the workspace of the paretic arm. Three sensors are attached to forearm, upper arm, and trunk to track arm movements in three-dimensional space while controlling for trunk compensation. Whole-arm movements serve as input for a therapy game. The reward group (*n* = 37) will train with performance feedback and contingent monetary reward. The control group (*n* = 37) uses the same system but without monetary reward and with reduced performance feedback. Primary outcome is the change in the hand workspace in the transversal plane. Standard clinical assessments are used as secondary outcome measures.

**Discussion:**

This randomized controlled trial will be the first to directly evaluate the effect of rewarding feedback, including monetary rewards, on the recovery process of the upper limb following stroke. This could pave the way for novel types of interventions with significantly improved treatment benefits, e.g., for conditions that impair reward processing (stroke, Parkinson’s disease).

**Trial registration:**

ClinicalTrials.gov, ID: NCT02257125. Registered on 30 September 2014.

**Electronic supplementary material:**

The online version of this article (doi:10.1186/s13063-017-2328-2) contains supplementary material, which is available to authorized users.

## Background

After stroke, 50% of survivors are left with impairments in arm function [1, 2], which is associated with reduced health-related quality of life [3]. While there is evidence for a positive correlation between therapy dose and functional recovery [4–6], a higher therapy dose is challenging to implement, as it usually leads to an increase in costs commonly not covered by health insurances. However, when dose is matched, most randomized controlled trials introducing new types of rehabilitative interventions (e.g., robot-assisted therapy [7]) failed to show a superior effect compared to standard therapy. Thus, the need for improving therapy effectiveness remains. In search for elements of effective therapy, we hypothesize that performance feedback and monetary rewards can improve effectiveness.

It has been shown that reward enhances procedural [8] and motor-skill learning [9, 10] and has a positive effect on motor adaptation [11]. Rewards mainly improve retention of motor skills and motor adaptations [9–11]. This effect was not explained by training duration (dose) as rewarded and non-rewarded groups underwent similar training schedules [8–11]. In a functional magnetic resonance imaging (fMRI) study, Widmer et al. reported that adding monetary rewards after good performance leads to better consolidation and higher ventral striatum activation than knowledge of performance alone [10]. The striatum is a key locus of reward processing [12], and its activity was shown to be increased by both intrinsic and extrinsic reward [13]. Being a brain structure that receives substantial dopaminergic input from the midbrain, ventral striatal activity can be seen as a surrogate marker for dopaminergic activity in the substantia nigra/ventral tegmental area [14]. In rodents, Hosp et al. found that dopaminergic projections from the midbrain also terminate directly in the primary motor cortex (M1) [15]. Dopamine in M1 is necessary for long-term potentiation of certain cortico-cortical connections and successful motor-skill learning [16]. As mechanisms of motor learning are also thought to play a role in motor recovery [17], rehabilitative interventions may benefit from neuroplasticity enhanced by reward.

Here, we describe a trial protocol to test the effect of enhanced feedback and reward on arm rehabilitation after stroke at matched training dose (time and intensity). We use the ArmeoSenso, a standardized virtual reality (VR)-based training system [18] that is delivered in two versions for two different study groups, one version with and one without reward and enhanced performance feedback.

## Methods

### Ethics and reporting

The study protocol follows the Consolidated Standards of Reporting Trials (CONSORT) Statement on randomized trials of non-pharmacological treatment [19] and Standard Protocol Items: Recommendations for Interventional Trials (SPIRIT; see Fig. 5 for the SPIRIT Figure and the SPIRIT Checklist in Additional file 1) guidance for protocol reporting [20]. The study is recruiting patients at three different rehabilitation clinics. The procedures and the protocol (version 4.1 of 18 August 2016) have been approved by the responsible Ethics Committees “Ethikkommission Nordwest- und Zentralschweiz,” the “Kantonale Ethikkommission Zürich” (LU2013-079 and PB_2016-01804) and the Swiss Agency for Therapeutic Products (Swissmedic: 2014-MD-0033) and conform to the guidelines of Good Clinical Practice E6 (R1). All subjects have to give written informed consent in accordance with the Declaration of Helsinki. A quality assurance audit/inspection of this study may be conducted by the competent authority or Ethical Committees, respectively. The quality assurance auditor/inspector will have access to all medical records, the investigator’s study-related files and correspondence, and the informed consent documentation that is relevant to this clinical study.

### Study design

**Fig. 1:**
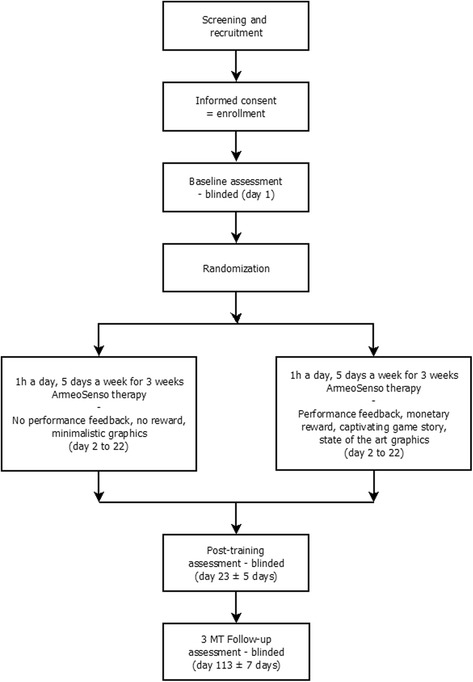
Flow diagram illustrating the trial design and sequence

This multicentric trial is randomized, controlled and assessor-blinded (Fig. 1). Patients are unaware of the training characteristics of the other study group.

### Study population

This study includes stroke patients (maximum 100 days after stroke) who meet the following criteria: minimum age of 18 years, hemiparesis of the arm due to cerebrovascular ischemia, the ability to lift the paretic arm against gravity, a minimal arm workspace of 20 cm × 20 cm in the horizontal plane, ability and willingness to participate, as well as the absence of severe aphasia (i.e., patients who are not able to follow two-stage commands), depression, dementia and hemianopia.

### Randomization

The randomization procedure was planned and set up by an independent contract research organization (Appletree CI Group, Winterthur, Switzerland). A non-consecutively increasing, pseudo-randomly generated list of subject identification numbers (IDs) was created. IDs are chronologically assigned to each new study participant, stratified by the study center. Allocation to one of the two study groups is balanced in blocks of 4. The randomization list containing the subject ID, the corresponding group allocation and a randomly generated password was sent to an independent (unblinded) study staff member (“admin”) who has set up respective patient-user computer accounts used for accessing the therapy game. The group-specific version of the game, i.e., either with or without reward, is defined by the account. The admin keeps the assignment list and is not involved in data collection.

Immediately before the first training session, each study participant has to confirm by signature to have received a sealed envelope containing a butterfly etiquette with ID and password to access the account. The patient keeps this etiquette for the entire study duration.

### ArmeoSenso training system

**Fig. 2:**
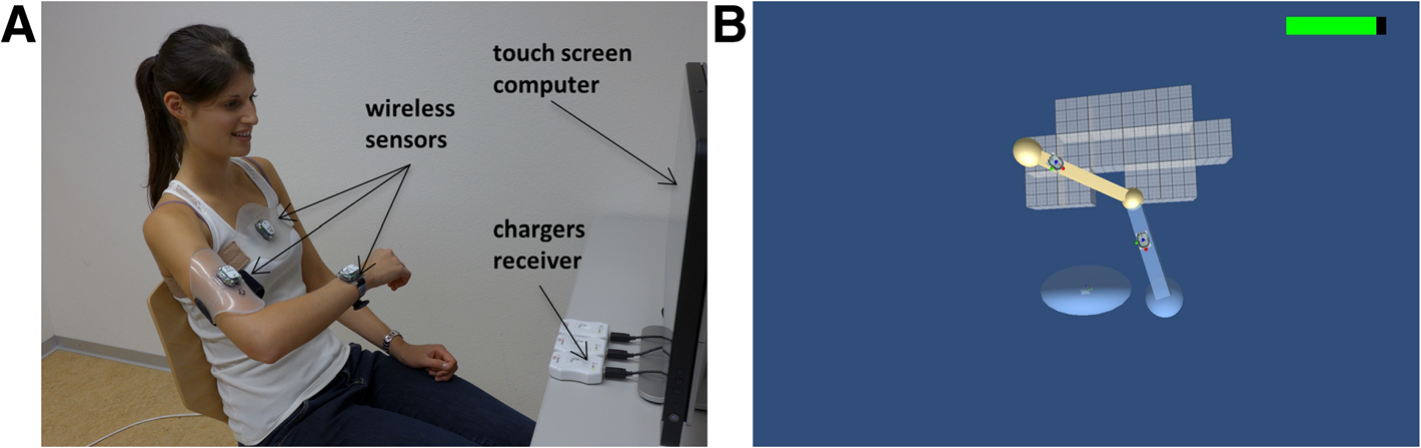
**a** Healthy subject using the ArmeoSenso training system. **b** Arm workspace assessment: *gray cubic* voxels arranged in the transverse plane reflecting 10 cm × 10 cm active workspace relative to the patient’s trunk

The arm rehabilitation system combines motion capturing via wearable inertial measurement units (IMUs) in combination with a therapy game, running on a touch screen computer (Inspiron 2330, Dell Inc., Round Rock, TX, USA) (Fig. 2a). Three wireless IMUs (MotionPod 3, Movea SA, Grenoble, France) are fixed to the functionally impaired lower and upper arm as well as the trunk [18, 21].

**Fig. 3:**
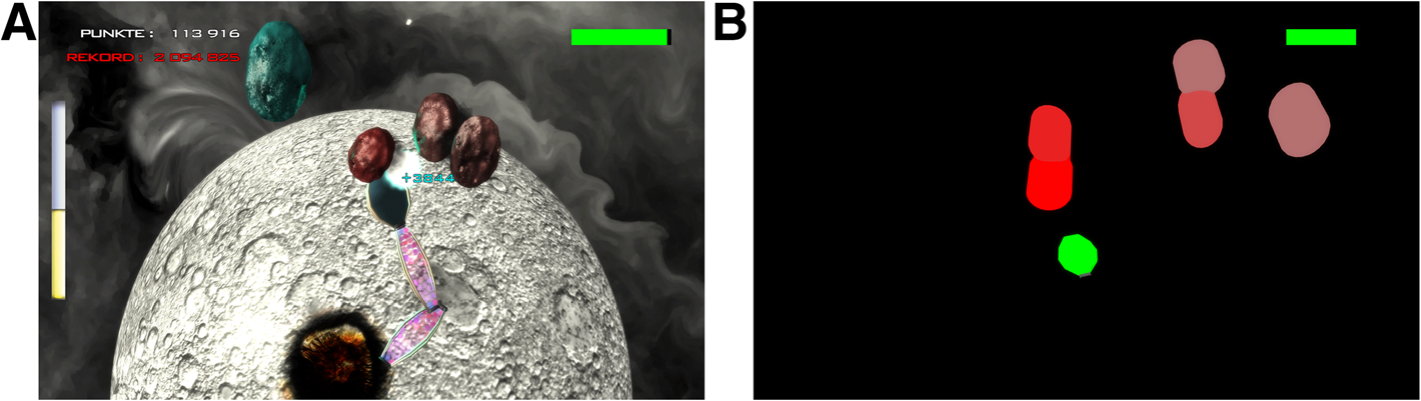
**a** ArmeoSenso-Reward: “METEORS” therapy game. The hand of the virtual arm is used to catch the falling meteors before they crash onto the planet. If caught, the meteor explodes and a score appears. If missed, the planet gets damaged (note the impact crater). The current score (= PUNKTE) is displayed on the upper left (*white font color*) and compared to the patient’ all-time record (= REKORD; *red font color*, *upper left*). The *green* bar on the *upper right* indicates resting time. If completely black, the patient has to rest for 4 s before new meteors are spawned. During rest, the bar fills with green. The *yellow bar* on the *left* indicates how much playtime is left in the ongoing round (maximum 150 s). **b** Control game. The virtual hand is a *green decagon* that can be used to touch the pill-shaped, single-colored targets dropping in from the top of the screen, which then disappear with a delay of 1 s without producing a score. The *green bar* on the upper *right* fills up whenever the patient assumes the resting position

In contrast to robot-based VR therapy systems, this sensor-based approach does not offer any weight support for the impaired arm. The ArmeoSenso system specifically requires the patient to lift the arm against gravity and to increase hand workspace in three-dimensional (3D) space. The system was validated in a home feasibility trial with stroke patients [21]. For the present study, the ArmeoSenso system includes two automated functional assessments, one consisting of a pointing task with nine targets arranged in two semicircles in the transversal plane. The second assessment measures the hand workspace of the trained limb (see the “Primary outcome” section). While identical assessments are performed in both training groups, the system includes a specific version of a therapy game for each of the two training groups: (A) a rewarding version including monetary rewards, knowledge of performance feedback and graphical special effects (Fig. 3a) and (B) a non-rewarding version lacking these motivators (Fig. 3b). A more detailed description follows.

### Intervention

In addition to standard therapy, both groups train for 1 h per day, 5 days a week for 3 weeks while inpatients in a participating rehabilitation hospital. Note that for study participants, standard therapy excludes additional proximal-arm training. All other therapies, however, are not affected by the study.

ArmeoSenso training is supervised by a therapist. Since 1 h of consecutive upper-limb training per day without weight support can be too demanding for some patients, deviations from this protocol are allowed to a minimum cumulative training time of 720 min.

A typical ArmeoSenso training session is described in Wittmann et al. [21]. For the present study, patients log in to their user account with their random ID and the password printed on their butterfly etiquette. The IMUs are fixed to the affected lower and upper arm and to the trunk using custom-made Velcro straps (Balgrist Tec AG, Zurich, Switzerland). The supervising therapist may help if necessary. The ArmeoSenso system then guides the patient through three calibration poses and two automated assessments (see the “Outcome measures” section) before training starts (beginning of the targeted 60-min session duration). In order to prevent physical exhaustion, the patient is visually instructed to rest for at least 4 s every 40 s. Moreover, patients are allowed to interrupt the training session if an additional break is needed. The duration of the additional breaks is added at the end of the training session. After 60 min of net training time, the automated assessments will be repeated and the patient will be asked to fill in a short motivation questionnaire (see the “Secondary outcome” section).

Both groups train with modified versions of the ArmeoSenso “METEORS” game (see [18, 21]). Although the two versions differ markedly in terms of their appearance, they share the underlying game mechanics. That is, in both a virtual “hand” which matches the movement of the subject’s real hand is used to catch objects that drop downwards from the top of the screen. The targets are placed within, or at the border of, the patient's virtual 3D workspace, which is continuously estimated and updated using a voxel-based model [18]. The time to complete a round in the METEORS game is T_max = 150 s (excluding rest). If, during these 150 s, less than five targets were missed, the round is won and the difficulty increases by up to three levels, depending on the number of targets that hit the ground. Difficulty is adapted dynamically by changing (1) the average target speed of falling, (2) the target spawn interval and (3) the number of simultaneously spawned targets (one to a maximum of seven). It increases in this order (i, ii, iii, i, …). Conversely, difficulty decreases in reverse order if more than four targets were missed and the round is lost after a certain time (T_loss). In that case, the difficulty decrease is calculated by rounding $\frac{T\_\mathit{\max}}{T\_ loss}$ to the closest integer, but with a maximum of four difficulty levels.

### Rewarded training

The reward group will train for 15 h with a version of the METEORS game that is very similar to the one used in previous studies [18, 21]. Briefly, the hand is used to catch the targets that are depicted as meteors. The movement of the patient’s whole arm is displayed with low latency on the computer screen as a moving virtual arm; a feature implemented to increase the feeling for embodiment and thus improve the motivation to move the arm [22]. Subjects are instructed to use the hand to catch the falling meteors in order to protect their planet from being destroyed (Fig. 3a). This game theme is easily understood and emotionally involving [21].

Whenever a meteor is touched by the virtual hand, it explodes, giving the patient immediate knowledge of the result. Furthermore, a score appears with each exploding meteor that depends on the falling speed and diminishes with the time the meteor was visible on the screen before being caught. Scores are summed up over a round and reset when the next round starts. However, there is also an all-time high score always visible on the upper left (Fig. 3a). If a meteor is missed, it crashes on the planet and damages it. Should the patient miss more than four meteors within T_loss < 150 s, the round is lost, which results in visual effects showing the planet being destroyed and the camera shaking, followed by a message encouraging the patient to try again.

**Fig. 4:**
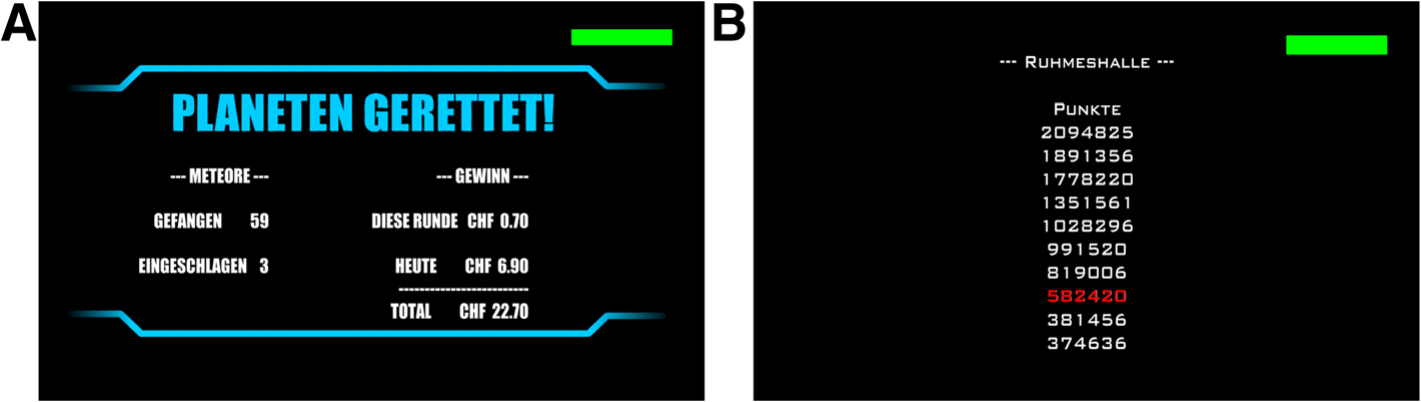
ArmeoSenso-Reward feedback screens. **a** “PLANETEN GERETTET”: planet saved. This screen is presented after each completed round. The number of meteors caught (“GEFANGEN”, *top*) and meteors hitting the planet (“EINGESCHLAGEN”, bottom) is indicated on the *left*. The monetary reward (“GEWINN”) for the current round (“DIESE RUNDE”, *top*), the current day (“HEUTE”, *middle*) and the total amount of money gathered over the course of the study (“TOTAL”, *bottom*) are displayed on the right. Note that a maximum of 1 Swiss Franc (CHF) can be won per round. **b** Hall of fame (“RUHMESHALLE”) with the patient’s top 10 scores. If the current score is in the top 10, it is highlighted in *red*

After successful level completion, patients are shown a feedback screen illustrating that they have successfully saved the planet, how many meteors they managed to catch and how many they have missed (Fig. 4a). Monetary rewards are given for each completed level. Patients can win up to 1 Swiss Franc (CHF; approx. US$20), if they succeed, but 0.1 CHF is deducted for every missed meteor. As a new level can be started approximately every 3 min, a maximum of 20 CHF could be won per training session in case of an uninterrupted winning streak. This, however, is unlikely due to the difficult adaptation described above. All of it, the money won during the preceding round, during the ongoing training session and the total money gathered over the whole course of the study, is presented on the feedback screen (Fig. 4a), which is followed by a high score list showing the top 10 results (Fig. 4b). If the current result was in the top 10, it is marked in the list (Fig. 4b). This feature was also implemented to optimize patient engagement.

New planets (eight in total) and/or backgrounds (12 in total) are unlocked during the course of the 3-week training. These rewards do not have any influence on the gameplay and difficulty but are intended to add variety to the game. Once three planets have been unlocked, the patient can choose between two randomly selected planets at the start of every round.

### Control training

The control training consists of the same sensor system and game mechanics with all rewarding feedback removed. In order to reduce the feeling of embodiment [22], only the position of the hand is shown as a green decagon on a plain black background. Targets are simple pill-shaped, single-colored objects that disappear with a delay of 1 s without producing a score or sound after being touched; hence, there is no immediate but delayed knowledge of performance. Complete removal of knowledge of performance is not possible in this game because patients then might reach for the same target several times, which would hamper comparability to the other study group. The feedback screen, the monetary reward, the high score list and the unlocking of new planets and backgrounds are also removed. Instead, patients are looking at a blank screen to keep the training time comparable. Most notably, the target placement and difficult adaptation remain unaffected.

### Outcome measures

**Fig. 5:**
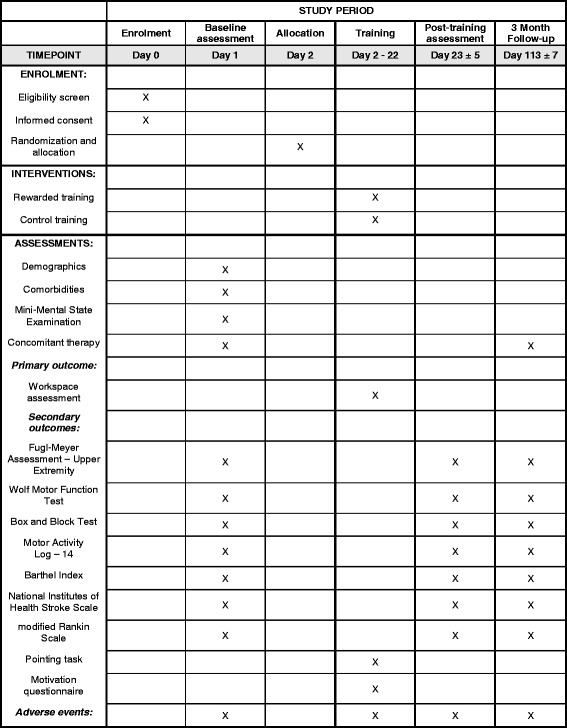
Standard Protocol Items: Recommendation for Interventional Trials (SPIRIT) Figure. The schedule of enrollment, interventions and assessments

The clinical assessments are collected by assessors blinded to treatment allocation. All assessors are trained in performing the assessments before the start of the trial. In addition to the outcome measures described below, demographics, comorbidities, cognitive function (Mini Mental State Examination) and concomitant therapy will be recorded (Fig. 5).

#### Primary outcome

The primary outcome of this trial is the workspace of the impaired arm in the horizontal plane, measured by using an assessment integrated into the ArmeoSenso platform. Subjects are instructed to actively reach out as far as possible with their impaired arm forward, backward and sideways to explore the entire arm workspace. The workspace is corrected for trunk movements and computed as the number of square pixels of 10-cm side length arranged in the transverse plane relative to the patient’s trunk (Fig. 2b) (see Wittmann et al. [18] and Wittmann et al. [21] for more information). This assessment is conducted immediately before and after every therapy session (Fig. 5).

#### Secondary outcome

Arm impairment is assessed using the Fugl-Meyer Assessment-Upper Extremity (FMA-UE), arm activity using the Wolf Motor Function Test (WMFT), the Box and Block Test and a pointing task (ArmeoSenso integrated assessment) (Fig. 5). For the pointing task, nine targets arranged in two semicircles appear one after another in the transversal plane in front of the subject. The goal is to reach out to the target within 8 s. The number of targets reached and the mean time to target is reported. The Motor Activity Log 14 (MAL-14) for self-reported movement ability, the Barthel Index (BI) as a measure of independence in daily living, the National Institutes of Health Stroke Scale (NIHSS) as a measure of stroke severity are recorded and the global disability is assessed using the modified Rankin Scale (mRS) (Fig. 5).

Finally, patients fill in a short questionnaire after each training session. Ten questions (five positively and five negatively formulated), given in randomized order, evaluate the subjective appraisal of the training on a five-point Likert scale (Fig. 5).

#### Assessments of safety

Adverse events (AEs) expected to occur are skeletal or muscular pain and fatigue indicating a syndrome of overuse. The quality management system of the Clinical Trial Center Zurich will be followed according to national and international guidelines [23]. Adverse events (AEs) will be documented and related serious adverse events (SAEs) will be reported to the Ethical Committee, the competent authority (Swissmedic) and local principle investigators (PIs). All SAEs will be included in an annual report to authorities and PIs. AEs will be recorded from baseline assessment to the end of the trial (Fig. 5).

### Sample size

The sample size is estimated to detect a between-group difference of 4.8 voxels in the workspace difference from beginning to end of training, based on the improvement in arm workspace from pilot results (unpublished) and an estimated group difference of 20%. This assumes a two-sided alpha level at .05 and a power of 80%. For an effect with a standard deviation of seven voxels, 35 subjects per group yields 80% power to detect the true alternative. We will randomize 37 subjects in each group, based on our observed attrition rate of 5% in a previous interventional trial [21]. This calculation was performed using G*Power 3.1 [24, 25].

### Statistical analysis

Our primary analysis is an intention-to-treat analysis comparing the two groups. In addition, as this is an explanatory trial, a per-protocol analysis will be used to analyze the effect of feedback under ideal conditions [26, 27]. Therapy will take place in 15 sessions over 3 weeks, and there is the possibility that some subjects will not complete the full treatment regimen due to scheduling issues or other time constraints. If they still perform at least 12 h of therapy the data will be analyzed. All other patients will be considered “non-compliant” in the sense that they do not receive the full treatment dose. According to the per-protocol principle, their outcomes will not be analyzed.

A two-sample *t* test comparing the mean change in voxel workspace assessment between the two groups will be used; in case of non-normality, a Mann-Whitney test will be computed instead. Moreover, repeated measures analysis of variance (ANOVAs), in case of normally distributed data, or non-parametric Friedman tests will be used to assess the development of the different outcome measures over time. Statistical significance will be based on a *p* value threshold of 0.05. Data will be analyzed using MATLAB R2013b (or newer) (MathWorks Inc., Natick, MA, USA) and SPSS (version 23 (or newer), IBM Corp., Armonk, NY, USA).

## Discussion

This is the first randomized clinical trial to evaluate the effect of enhanced feedback and reward on arm rehabilitative training following stroke. Intrinsic (score, knowledge of performance) and extrinsic rewards (money) hypothetically improve motor cortex plasticity and overall motivation to train. Because motivation affects training time and time is a crucial determinant of effect [4], this trial controls for time by using a control intervention that is matched in time and dose of training.

In a motor learning study with healthy young subjects, we have shown that the consolidation/retention of a skilled motor task is more effective if the task was trained in the presence of reward [10]. In a rat model, projections from midbrain dopaminergic regions to M1 are required for successful motor learning and functional plasticity at cortical (layer II/III) synapses [15, 16], mechanisms that presumably support recovery after stroke [28]. Whether the dopaminergic system can be stimulated to improve recovery remains to be shown. Likewise, whether reward is an appropriate stimulus is yet unknown.

Previous studies have assessed the patient’s motivation for a specific training (e.g., Wittmann et al. [21] and Nijenhuis et al. [29]), but none of them compared the outcome to an appropriate control condition for the evaluation of the effectiveness of rewarding therapy. Although functional improvement itself might be motivating enough for some patients to train, here we are in search of a clinical effect of reward on a reduction in impairment (shoulder/elbow range of motion (ROM)) mediated by active and repetitive proximal-arm training. We chose this training method because (1) it can be standardized in its conduct and has quantifiable parameters of dose, movement success and arm workspace as primary outcome measure, (2) it is based on a therapy system which was already evaluated with patients and found to be safe, (3) it is easily supported in participating institutions without much training of therapists who provide assistance to the patient and (4) it has shown a moderate effect on chronically arm-impaired stroke survivors [21]. Because the ArmeoSenso training only works on proximal-arm function, it is not expected to have a clinically relevant effect on activities of daily living, independence, or quality of life. We therefore chose a primary outcome that is close to what is actually being trained, i.e., arm workspace. Workspace assessments have been widely used to assess the arm function of stroke patients, thereby showing high correlation to standard clinical scales [30, 31]. For a discussion of clinical relevance of functional outcomes, in the interest of clarity and conciseness, we would like to refer to the review by Ashford et al. [32]. Nevertheless, potential transfer to more clinical scores can be tracked using our secondary outcome measures.

The study is enrolling subjects during the initial 3 months after stroke. Most recovery is occurring in this period [33–37]. Therefore, we expect an improvement in arm function in both groups.

A positive outcome of this trial will emphasize the role of reward in rehabilitative training. This result could potentially be applicable to various forms of post-stroke rehabilitative training. Social rewards (smileys, praise), food rewards (sweets, dietary allowance), monetary reward or token programs are options that are easy to implement in situations where there is systematic interaction between a patient and a human trainer or a technical training device. Virtual-reality-based training games, therapy elements including repetitive performance feedback and similar approaches are examples where to integrate reward according to suggestions to be derived from the described study.

### Trial status

The trial is currently recruiting patients. At the time of submission, 16 stroke patients have been enrolled.

## Additional files

Additional file 1: SPIRIT 2013 Checklist. (DOC 121 kb)

Additional file 2: Informed Consent Form (German). (PDF 331 kb)

## Abbreviations

AEs: Adverse events
BI: Barthel Index
EKNZ: Ethikkommission Nordwest- und Zentralschweiz
FMA-UE: Fugl-Meyer Assessment of the upper extremity
GCP: Good Clinical Practice
IDs: Identification numbers
IMU: Inertial measurement unit
M1: Primary motor cortex
MAL14: Motor Activity Log 14
mRS: modified Rankin Scale
NIHSS: National Institutes of Health Stroke Scale
PIs: Principle investigators
RCT: Randomized controlled trial
ROM: Range of motion
SAEs: Serious adverse events
VR: Virtual reality
WMFT: Wolf Motor Function Test
