# Supplementary material for: Does motivation matter in upper-limb rehabilitation after stroke? ArmeoSenso-Reward: study protocol for a randomized controlled trial
Source: Trials. 2017 Dec 2;18:580. doi: 10.1186/s13063-017-2328-2 (PMC5712159; doi:10.1186/s13063-017-2328-2)
Supplement: Supplementary file 2 — Informed Consent Form (German). (PDF 331 kb) [file 13063_2017_2328_MOESM2_ESM.pdf]

Zürcher RehaZentren

Wald, 13. Juli 2016

Teilnehmerinnen-/Teilnehmerinformation

ArmeoSenso – therapy for patients with arm impairments based on wearable movement sensors

Sponsor: Prof. Dr. med. A. Luft, Universität Zürich

Sehr geehrte Teilnehmerin, sehr geehrter Teilnehmer

1. Auswahl der StudienteilnehmerInnen

Wir fragen Sie an, ob Sie an dieser Studie teilnehmen möchten, weil Sie wegen eines Schlaganfalles an einer Lähmung eines oder beider Arme leiden. In dieser Studie soll die Wirksamkeit eines für die Heimtherapie konzipierten Therapiesystems (Name: ArmeoSenso) zur Verbesserung der Armfunktion, bestehend aus einem Computer und aus tragbaren Bewegungssensoren, getestet werden. Zwecks Vergleichbarkeit der Daten wird das Training von Ihren Therapeuten überwacht und findet deshalb hier im Zürcher RehaZentrum Wald statt.

2. Ziel der Studie

Das Ziel der Studie ist es, die Wirksamkeit verschiedener Versionen dieses Therapiesystems zu erfassen und miteinander zu vergleichen. Dabei trainieren Sie, vergleichbar mit der Standardtherapie, gezielt die Erweiterung des Bewegungsbereichs im 3D Raum, die Muskulatur, die Feinmotorik, und die Koordination des gesamten Armes mit dem Ziel, die Armfunktion zu verbessern. Es handelt sich bei den zu testenden Therapiesystemen um Prototypen. Die Erkenntnisse aus dieser Studie tragen massgebend zu einer möglichst effektiven Gestaltung der ArmeoSenso-Therapie bei.

3. Allgemeine Informationen zur klinischen Studie

- Rehabiliationstherapie nach einem Schlaganfall kann die Funktionsfähigkeit auch Monate und Jahre nach einem Schlaganfall verbessern. Da die Therapie jedoch teuer und aufwändig ist und durch die Krankenkassen oft nicht im gewünschten Umfang bezahlt wird, haben wir ein Heimtherapiesystem entwickelt. Dieses Heimtherapiesystem heisst „ArmeoSenso“ und soll ohne Hilfe durch einen Therapeuten zu Hause angewendet werden können. Dies ermöglicht ein häufiges und intensives Trainieren zu jedem beliebigen Zeitpunkt von beliebiger Dauer. Für die Erfassung der Wirksamkeit des ArmeoSenso-Therapiesystems wird in Rahmen dieser Studie das Training überwacht und die Dosis vorgegeben, was eine Durchführung der Studie direkt am Rehabilitationszentrum (Zürcher RehaZentrum Wald) bedingt.

- Das Trainingssystem besteht aus einem Computer mit Trainingssoftware, welche durch tragbare Sensoren gesteuert wird. Die Sensoren werden am Handgelenk, Oberarm und Rumpf getragen und senden die aufgezeichneten Bewegungsinformationen an den Computer, wodurch Rehabilitationsspiele interaktiv gesteuert werden. Durch spannende und unterhaltende Trainingsbedingungen sollen die Patientinnen/Patienten zu guten Leistungen motiviert werden.

- Ein ArmeoSenso Training läuft wie folgt ab: Vor jedem Training befestigen Sie 3 Bewegungs-Sensoren alleine oder mit Hilfe Ihres Therapeuten an Ihrem Handgelenk, Oberarm und Oberkörper. Danach starten Sie die Trainingstherapie, wobei Sie vom Computerprogramm durch das ganze Training geführt werden. Vor und nach jedem Training findet eine kurze automati-

sierte Prüfung der Armfunktion statt. Auch hier werden sie vom Computer durch alle notwendigen Schritte geführt. Nach dem Training ziehen Sie die Sensoren wieder aus und setzten sie bis zum nächsten Gebrauch auf die Ladestation.

- Insgesamt sollen 74 Patientinnen/Patienten mit einem Schlaganfall in die Studie eingeschlossen und einer von 2 Gruppen zugeteilt werden. Die Studie findet für Sie ausschliesslich hier im Zürcher RehaZentrum Wald statt. Zwecks Vergleichbarkeit der Daten muss, auch wenn für die Heimtherapie konzipiert, das Training jeweils überwacht und deshalb im Rehabilitationszentrum durchgeführt werden.

- In der Studie erhalten alle TeilnehmerInnen bezüglich Intensität und Umfang vergleichbare Therapie und es gibt keine Scheinbehandlung. Alle andern Therapien (Logo, Neuropsychologie, Gangtherapie, etc.) sind von einer Studienteilnahme nicht betroffen und werden unverändert fortgeführt. Falls Sie aktuell irgendeine Form von Therapie erhalten (Physio, Ergo, Logo, MTT) hat dies keinen Einfluss auf die Studie. Wir bitten Sie jedoch, dies uns mittzuteilen.

- Falls Sie sich für eine Teilnahme an dieser Studie entscheiden, bitten wir Sie nicht mit anderen StudienteilnehmerInnen über die Einzelheiten der ArmeoSenso-Therapie zu sprechen. Nur so können wir sicherstellen, dass die Wirkung der Therapie, die teils auf Motivation und Einstellung beruht, bei unterschiedlichen Teilnehmern unabhängig untersucht werden kann.

- Diese Studie wird in Übereinstimmung mit der schweizerischen Gesetzgebung und nach international anerkannten Richtlinien durchgeführt. Sie wurde von der zuständigen, unabhängigen Ethikkommission des Kantons genehmigt.

4. Freiwilligkeit der Teilnahme

Ihre Teilnahme an dieser Studie ist freiwillig. Wenn Sie nicht an dieser Studie teilnehmen wollen, haben Sie keine Nachteile für Ihre medizinische Betreuung zu erwarten. Das Gleiche gilt, wenn Sie Ihre einmal gegebene Einwilligung zu einem späteren Zeitpunkt widerrufen. Diese Möglichkeit haben Sie jederzeit. Einen allfälligen Widerruf Ihrer Einwilligung bzw. den Rücktritt von der Studie müssen Sie nicht begründen. Im Fall eines Widerrufs werden die bis zu diesem Zeitpunkt erhobenen Daten weiterverwendet.

5. Studienablauf

Die Studie dauert für Sie insgesamt ca. 4 Wochen. Während 3 Wochen trainieren Sie während 5 Stunden pro Woche ihre Armfunktion mit dem ArmeoSenso-Trainingssystem. Am Anfang werden Sie über die Funktion instruiert. Ansonsten sollen Sie das Training so selbständig wie möglich durchführen. Es wird jedoch immer ein Therapeut oder Bewegungswissenschaftler anwesend sein, um Ihnen, wenn nötig, behilflich zu sein und das Training zu überwachen.

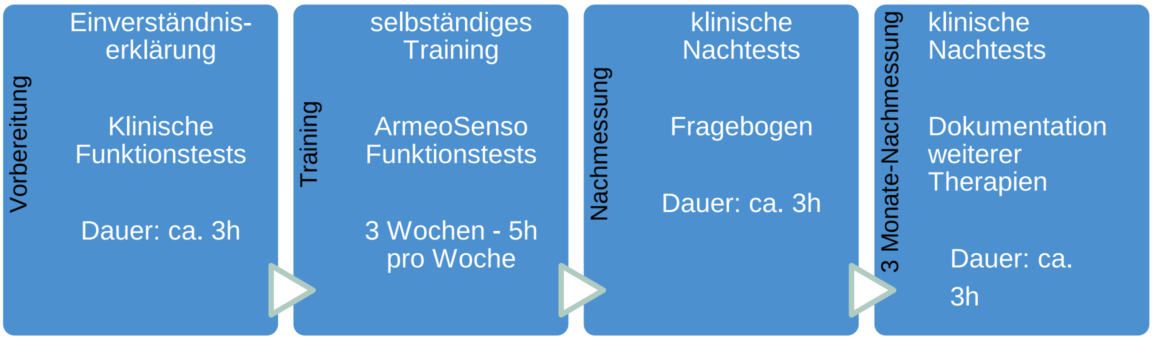

Abb. 1: Die 4 Phasen dieser Studie. Anhand von klinischen Tests, welche vor, gleich nach und 3 Monate nach dem Training durchgeführt werden, können wir die Wirksamkeit der ArmeoSenso-Therapie bewerten.

In der Vorbereitungs-, Nachmessungs- und 3 Monate-Nachmessungsphase werden verschiedene klinischen Funktionstests durchgeführt, die zusammen jeweils nicht mehr als 3 Stunden dauern sollten. Dazu gehören 3 Funktionstests, welche den Schweregrad der Armfunktionsstörung erfassen, 3 Tests, welche den Schweregrad des Schlaganfalles bewerten und ein Test, welcher allfällige kognitive Einschränkung bei einem Schlaganfall erfasst. Zudem wird mit dem MAL (einem strukturierten Interview) der tägliche Armeinsatz erfragt. Zur 3-Monate-Nachmessung gehört auch, dass dokumentiert wird, welche Therapien Sie seit Studienende in welchem Ausmass genossen haben. Ausserdem werden Sie nach jedem Training einen Fragebogen ausfüllen, welcher Ihre Motivation, mit dem ArmeoSenso-Therapiesystem zu trainieren, abfragt.

Während des Trainings zeichnet der Computer die Trainingsdaten (Trainingsdauer, Trainingsintensität, Bewegungsqualität) kontinuierlich auf und speichert sie auf der Festplatte. Dazu werden jeweils vor und nach dem Training ins Programm eingebaute Funktionstests durchgeführt, welche Ihre Armfunktion und allfällige Fortschritte über die Trainingsphase hinweg aufzeichnen.

6. Pflichten des Studienteilnehmers/Studienteilnehmerin

Als StudienteilnehmerIn sind Sie verpflichtet,

I. den medizinischen Anweisungen des Studienleiters zu folgen und sich an den Studienplan zu halten.

II. Ihren Studienarzt über den Verlauf der Erkrankung zu informieren und neue Symptome, neue Beschwerden und Änderungen im Befinden zu melden.

III. Ihren Studienarzt über gleichzeitige andere Therapien, die nicht im Zürcher RehaZentrum Wald durchgeführt werden, (Physiotherapie, Ergotherapie oder Logopädie) zu informieren.

7. Andere Behandlungsmethoden

Bitte beachten Sie, dass eine Studienteilnahme erfordert, dass Sie über die Studiendauer hinweg keine andere Armtherapie zur Erweiterung des Bewegungsspielraums im 3D-Raum als die in diesem Studienplan vorgeschlagene in Anspruch nehmen. Alle andern Therapien (Logo, Neuropsychologie, Gangtherapie, etc.) sind von einer Studienteilnahme nicht betroffen und werden unverändert fortgeführt.

8. Nutzen für die TeilnehmerInnen

Die Teilnahme an dieser klinischen Studie könnte Ihnen folgenden Nutzen bringen:

- Das Trainieren mit dem ArmeoSenso Trainingssystem könnte zu einer Verbesserung der Armfunktion führen. Dies ist bisher aber nicht gesichert.

- Ihre Teilnahme kann auch zur Verbesserung der Behandlung bei anderen Schlaganfallpatienten beitragen.

9. Risiken und Unannehmlichkeiten

Nebenwirkungen des verwendeten Medizinalproduktes sind nicht bekannt. Es gibt möglicherweise Risiken, die zum jetzigen Zeitpunkt nicht bekannt sind. Neu auftretende Symptome und Beschwerden sind dem Studienarzt zu melden.

Für Frauen, die schwanger werden können

Eine mögliche Schwangerschaft wird durch die Studienteilnahme nicht beeinträchtigt und ist kein Ausschlussgrund.

10. Neue Erkenntnisse

Der Studienarzt wird Sie während der Studie über alle neuen Erkenntnisse informieren, die den Nutzen der Studie oder ihre Sicherheit und somit Ihre Einwilligung zur Teilnahme an der Studie beeinflussen können. Sie werden die Information mündlich und schriftlich erhalten.

11. Vertraulichkeit der Daten

In dieser Studie werden persönliche und medizinische Daten von Ihnen erfasst. Diese Daten werden verschlüsselt, d.h. mit einem Code versehen. Diese Codeliste wird beim Prüfarzt aufbewahrt. Nur verschlüsselte Daten sind den Fachleuten zur wissenschaftlichen Auswertung zugänglich. Spezielle Fachleute des Sponsors (oder deren Beauftragte) können im Rahmen von Qualitätskontrollen die Durchführung der Studie überprüfen. Diese, sowie im Rahmen von Inspektionen auch die Mitglieder der zuständigen Behörden und Ethikkommissionen können über Ihren Studienarzt Einsicht in Ihre nicht codierte Krankengeschichten nehmen. Im Schadenfall erhalten Vertreter der Versicherung ebenfalls über Ihren Studienarzt Einsicht in Ihre medizinischen Daten, jedoch nur soweit dies zur Erledigung des Schadenfalles notwendig ist. Während der ganzen Studie und bei den erwähnten Kontrollen wird die Vertraulichkeit strikt gewahrt. Ihr Name wird in keiner Weise in Rapporten oder Publikationen, die aus der Studie hervorgehen, veröffentlicht.

Verantwortlich für die Einhaltung der nationalen und internationalen Richtlinien zum Datenschutz ist der Sponsor in der Schweiz.

12. Kosten

Die in dieser Teilnehmerinformation erwähnten studienspezifischen Untersuchungen bzw. Studienmedikamente sind kostenlos. Weder Ihnen noch ihrer Krankenkasse entstehen im Zusammenhang mit Ihrer Teilnahme zusätzliche Kosten. Medikamente und Therapien, die Sie unabhängig von der Studie einnehmen oder verwenden, werden nicht vom Sponsor dieser Studie übernommen.

13. Unfreiwilliger Studienabbruch

Ihre Teilnahme kann durch den Studienarzt oder den Studiensponsor abgebrochen werden. Folgende Gründe können dazu führen:

I. Deutliche Verschlechterung der Armfunktion während der Studie, ohne dass eine eindeutige studienunabhängige Ursache zu eruieren ist.
II. Unerwartete unerwünschte Nebenwirkungen.
III. Nichteinhalten des Studienplans oder der medizinischen Anweisungen des Studienleiters.

14. Deckung von Schäden

Falls Sie durch die Studie einen Schaden erleiden, haftet die Institution oder Firma (der Sponsor), die die Studie veranlasst hat und für die Durchführung verantwortlich ist. Die Voraussetzungen und das Vorgehen sind gesetzlich geregelt. Im Auftrag der Universität Zürich hat die Cereneo AG, Seestrasse 18, 6354 Vitznau daher eine Versicherung bei der Chubb Insurance Company of Europe SE, Zollikerstrasse 141, 8034 Zürich abgeschlossen, um im Schadenfall für die Haftung aufkommen zu können. Wenn Sie einen Schaden erlitten haben, so wenden Sie sich bitte an den Prüfarzt oder an das oben erwähnte Versicherungsunternehmen.

15. Kontaktpersonen

Bei Unklarheiten, Notfällen, unerwarteten oder unerwünschten Ereignissen, die während der Studie oder nach deren Abschluss auftreten, können Sie sich jederzeit an die untenstehende Kontaktperson wenden:

Verantwortliche Studienärztin:	PD Dr. med. Antonella Palla<br>Zürcher RehaZentrum Wald<br>Faltigbergstrasse 7<br>8636 Wald<br>Tel.: 055 256 68 21<br>E-Mail: antonella.palla@zhreha.ch
Mitarbeiter:	Mario Widmer, MSc<br>Neurologische Klinik, Universitätsspital Zürich<br>Frauenklinikstrasse 26<br>8091 Zürich<br>Tel.: 044 255 88 06<br>E-Mail: widmemar@student.ethz.ch

Schriftliche Einverständniserklärung des Patienten zur Teilnahme an einer klinischen Studie

- Bitte lesen Sie dieses Formular sorgfältig durch.
- Bitte fragen Sie, wenn Sie etwas genauer wissen möchten oder nicht verstehen.

NUMMER DER STUDIE:	EKNZ (LU) 13079
TITEL DER STUDIE:	ArmeoSenso – therapy for patients with arm impairments based on wearable movement sensors
**Sponsor** (vollständige Adresse):	Prof. Dr. med. Andreas Luft, Universitätsspital Zürich, Neurologische Klinik, Frauenklinikstrasse 26, CH-8091 Zürich<br>Tel.: 044 255 54 00, Fax: 044 255 45 07, E-Mail: andreas.luft@usz.ch
ORT DER STUDIE:	Zürcher RehaZentrum Wald, Faltigbergstrasse 7, 8636 Wald
**Prüfer/Prüferin:**<br>Name und Vorname:	
**Probandin/Proband**<br>Name und Vorname:<br>Geburtsdatum:	☐ männlich ☐ weiblich

- Ich wurde vom unterzeichnenden Prüfer mündlich und schriftlich über die Ziele, den Ablauf der Studie mit dem ArmeoSenso-Therapiesystem, über die zu erwartenden Wirkungen, über mögliche Vor- und Nachteile sowie über eventuelle Risiken informiert.
- Ich habe die zur oben genannten Studie abgegebene schriftliche Probandeninformation vom 13.07.2016 gelesen und verstanden. Meine Fragen im Zusammenhang mit der Teilnahme an dieser Studie sind mir zufriedenstellend beantwortet worden. Ich kann die schriftliche Probandeninformation behalten und erhalte eine Kopie meiner schriftlichen Einverständniserklärung.
- Ich hatte genügend Zeit, um meine Entscheidung zu treffen.
- Ich bin darüber informiert, dass eine Versicherung Schäden deckt, falls solche im Rahmen der Studie auftreten.
- Ich weiss, dass meine persönlichen Daten nur in anonymisierter Form an aussenstehende Institutionen zu Forschungszwecken weitergegeben werden. Ich bin einverstanden, dass die zuständigen Mitarbeiter des Studienauftraggebers, der Behörden und der Kantonalen Ethikkommission zu Prüf- und Kontrollzwecken in meine Originaldaten Einsicht nehmen dürfen, jedoch unter Einhaltung der Vertraulichkeit.
- Ich nehme an dieser Studie freiwillig teil. Ich kann jederzeit und ohne Angabe von Gründen meine Zustimmung zur Teilnahme widerrufen. In diesem Fall werde ich zu meiner Sicherheit abschliessend medizinisch untersucht.
- Ich bin mir bewusst, dass während der Studie die in der Probandeninformation genannten Anforderungen und Einschränkungen einzuhalten sind.
- Im Interesse meiner Gesundheit kann mich der Prüfer jederzeit von der Studie ausschliessen. Zudem orientiere ich den Prüfer über die Behandlung bei einem anderen Arzt sowie über die Einnahme von Medikamenten (vom Arzt verordnete oder selbständig gekaufte). **b.w.**

Ort, Datum	Unterschrift der Probandin/des Probanden

Bestätigung des Prüfers: Hiermit bestätige ich, dass ich diesem Probanden/dieser Probandin Wesen, Bedeutung und Tragweite der Studie erläutert habe. Ich versichere, alle im Zusammenhang mit dieser Studie stehenden Verpflichtungen zu erfüllen. Sollte ich zu irgendeinem Zeitpunkt während der Durchführung der Studie von Aspekten erfahren, welche die Bereitschaft des Probanden/der Probandin zur Teilnahme an der Studie beeinflussen könnten, werde ich ihn/sie umgehend darüber informieren.

Ort, Datum	Unterschrift der Prüferin/ des Prüfers
